# Clinicopathological features and outcomes in patients with concurrent medullary and papillary thyroid carcinoma

**DOI:** 10.3389/fendo.2025.1625989

**Published:** 2025-08-11

**Authors:** Ruonan Sun, Xueting Liu, Jiabin Liu, Zhihui Li, Yichao Wang

**Affiliations:** ^1^ Division of Thyroid Surgery, Department of General Surgery, West China Hospital, Sichuan University, Chengdu, Sichuan, China; ^2^ West China School of Medicine, West China Hospital, Sichuan University, Chengdu, Sichuan, China; ^3^ Department of Evidence-Based Medicine and Clinical Epidemiology, West China Hospital, Sichuan University, Chengdu, Sichuan, China; ^4^ Department of General Surgery, Shangjin Nanfu Hospital, Chengdu, Sichuan, China; ^5^ Laboratory of Thyroid and Parathyroid Diseases, Frontiers Science Center for Disease-Related Molecular Network, West China Hospital, Sichuan University, Chengdu, Sichuan, China

**Keywords:** medullary thyroid carcinoma, papillary thyroid carcinoma, concurrent, prognosis, thyroid cancer

## Abstract

**Objective:**

The co-existence of medullary thyroid carcinoma (MTC) and papillary thyroid carcinoma (PTC) is rare. The study analyzed the clinicopathological findings and prognosis of concomitant PTC in MTC patients.

**Methods:**

Clinicopathological data and follow-up outcomes of 25 patients with concurrent medullary and papillary thyroid carcinoma (combination group) between January 2009 and May 2024 were collected and analyzed retrospectively. We compared clinicopathologic characteristics and follow-up outcomes between patients with concurrent MTC and PTC (combination group) and those with MTC alone (MTC group).

**Results:**

The 25 patients with concurrent MTC and PTC comprised 19 females and 6 males. There were no statistically significant differences between the combination group and the MTC group in terms of age, gender, or pathological features such as the diameter of MTC lesions, multifocality, extra-thyroidal extension (ETE), number of lymph node (LN) resected, the number of LN metastasis, the maximum diameter of LN metastasis, and TNM staging. The recurrence rate was similar between the two groups. Univariate analysis showed that the max tumor diameter, capsule invasion, extracapsular invasion and recurrent nerve invasion were associated with the risk of biochemical/structural abnormalities in MTC group. Multivariate analysis showed that only the max tumor diameter and capsule invasion were significant independent prognostic factors for biochemical/structural abnormalities.

**Conclusion:**

The result of this comparative study between patients with MTC and PTC co-existence and those with MTC alone showed similar invasiveness and prognosis.

## 1 Introduction

Thyroid cancer is the most common endocrine tumor, with a globally increasing incidence (1–4). The cancer cells originate from follicular cells and parafollicular C cells. The former gives rise to differentiated thyroid cancers, which include papillary thyroid carcinoma (PTC) and follicular thyroid carcinoma, as well as undifferentiated thyroid cancer (5–8). The latter refers to medullary thyroid carcinoma (MTC), a type of neuroendocrine tumor (9). Although originating from different cells, cases of a patient having both types of tumors simultaneously have been reported, though they are rare (<0.5%) (10, 11). Among these cases, the simultaneous occurrence of MTC and PTC is relatively more common and is usually reported in two forms. One is the mixed MTC-PTC, where a single tumor lesion contains both MTC and PTC components. The other is MTC with PTC, where the thyroid contains two separate tumor lesions.

Previous studies are case reports or case series (12–15). In particular, there is no large-scale comparative analysis of the clinical characteristics of concurrent MTC and PTC, nor comprehensive assessment of how these combined cases differ biologically from solitary MTC cases (11, 13, 16). To assess the biological aggressiveness of patients with concurrent MTC and PTC, we retrospectively reviewed clinical features of patients with concurrent MTC and PTC treated in our hospital. In addition, we compared clinicopathological features and outcomes between patients with MTC in conjunction with PTC and those with MTC alone.

## 2 Materials and methods

### 2.1 General information

A retrospective analysis was conducted on the clinical data of patients with medullary thyroid carcinoma (MTC) treated for the first time at our institution from January 2009 to May 2024. Patients with a history of or concurrent other malignancies were excluded. All patients underwent preoperative ultrasound-guided fine-needle aspiration biopsy, which indicated MTC or suspicious for MTC. Postoperative pathological examination confirmed MTC with combined PTC as the combination group, while those with only MTC were categorized into the MTC group. A total of 168 patients were included in the study, with an average age of (46.19 ± 14.43) years. Among them, there were 60 females and 99 males. The combination group comprised 26 patients with an age of 47.93 ± 8.56 years, while the MTC group included 142 patients with a mean age of 45.82 ± 15.41 years. Patients were followed up by telephone interviews and/or outpatient interview. The study was approved by the ethics committee of the West China Hospital of Sichuan University.

### 2.2 Surgical approach

Surgical treatment was based on the American Thyroid Association guidelines for MTC (17). The standard surgical approach was total thyroidectomy (TT) combined with bilateral central lymph node dissection (BCND). Ipsilateral lymph node dissection (ILND, levels II–V) was indicated if preoperative imaging showed no neck lymph node (LN) metastasis or distant metastasis, but serum calcitonin (Ctn) levels were >20 ng/L, or if preoperative imaging showed metastasis in the ipsilateral neck nodes with no contralateral lymph node involvement and serum Ctn ≤200 ng/L. Bilateral lymph node dissection (BLND, levels II–V) was indicated if preoperative imaging showed metastasis in the ipsilateral neck nodes with no contralateral lymph node involvement and serum Ctn >200 ng/L, or if preoperative imaging indicated bilateral neck lymph node metastasis or distant metastasis. In the combination group, preoperative biopsy results all suggested MTC or suspicion for MTC, with postoperative pathology revealing simultaneous PTC. The PTC lesions ranged from 1 to 6 mm in diameter, with an average diameter of (2.8 ± 1.6 mm). In 25 patients, MTC and PTC lesions were in different thyroid lobes, and in one case, the MTC lesion was located in ectopic thyroid tissue. In another case, both cancer lesions were in the same lobe but were distinct lesions. All 25 patients had lymph node metastasis from MTC, and 5 patients had ipsilateral central lymph node metastasis of PTC.

### 2.3 Follow-up and prognosis

Patients in the MTC group received levothyroxine replacement therapy postoperatively, while those in the combination group were given thyroid stimulating hormone (TSH) suppression therapy. Follow-up assessments were conducted at 1, 3, and 6 months postoperatively. Follow-up data were collected by telephone or outpatient interview. At 6 months post-surgery, patients with normal serum Ctn levels were followed annually, while those with elevated serum Ctn but ≤150 ng/L were followed every 6 months. Patients with serum Ctn >150 ng/L were followed every 3 months. Routine follow-up included serum Ctn, carcinoembryonic antigen (CEA), thyroid function tests, and neck ultrasound. For patients with serum Ctn >150 ng/L, chest and abdominal enhanced CT and pelvic or axial bone scintigraphy or whole-body PET-CT were added every 6 months. Prognosis was categorized into three types: disease-free survival, biochemical abnormality, and structural abnormality. Disease-free survival was defined as normal serum Ctn levels, negative Ctn stimulation tests, and no imaging abnormalities during follow-up. Biochemical abnormality was defined as elevated serum Ctn stable within a certain range, negative Ctn stimulation tests, and no imaging abnormalities. Structural abnormality was defined as elevated serum Ctn and/or positive stimulation tests with imaging evidence of disease. All patients with structural abnormalities underwent additional surgical treatment.

### 2.4 Statistical analysis

Data were analyzed using the Statistical Package for the Social Sciences (SPSS Inc, Chicago, IL, version 26.0). Categorical variables were analyzed using the Chi-square test, while continuous variables are expressed as the mean ± standard deviation (SD) were analyzed using the t-test. A univariate logistic regression was performed to evaluate the influence of each potential factors on the presence of recurrence or metastasis. The Kaplan-Meier method and the log rank test were used to estimate and compare differences between groups, Multivariate analysis was performed by Cox-Hazard regression model. In general, P-value <0.05 was considered statistically significant.

## 3 Results

### 3.1 Comparison of general information between the two groups

The demographic characteristics, such as the age, disease duration, presence of concurrent thyroid diseases, and surgical treatments between patients with MTC in conjunction with PTC and those with MTC alone, are compared in **Table 1**. There were no statistically significant differences among these characteristics. The postoperative hospital stay was also similar between the two groups.

**Table 1:** Basic data of the MTC+PTC group and the MTC group.

	MTC+PTC group (n=25)	MTC group (n=134)	X² (t)	P
Sex			2.418	0.12
Male	6 (24.0%)	54 (40.3%)		
Female	19 (76.0%)	80 (60.0%)		
Age (Mean ± SD)	47.93 ± 8.56	45.82 ± 15.41	-0.07	0.944
<55 years old	19 (76.0%)	95 (70.9%)	0.145	0.704
>55 years old	6 (24.0%)	39 (29.1%)		
HD	11	3	0.334	0.564
Surgery			3.295	0.193
TT+BCND	6 (24.0%)	33 (24.6%)		
TT+BCND+ILND	17 (68.0%)	64 (47.8%)		
TT+BCND+BLND	2 (8.0%)	17 (12.7%)		

SD, Standard deviation; MTC, Medullary thyroid carcinoma; PTC, Papillary thyroid carcinoma; HD, Hashimoto’s disease; TT, total thyroidectomy; BCND, bilateral central lymph node dissection; ILND, Ipsilateral lymph node dissection; BLND, Bilateral lymph node dissection.

### 3.2 Comparison of pathological data

In the combination group, the average maximum diameter of MTC lesions was no larger than that in the MTC group [(20.00 ± 13.36) mm vs. (25.32 ± 14.94) mm], but the difference was not statistically significant (P = 0.162). The combination group had 9 cases of multifocal MTC, whereas the MTC group had 44 cases of multifocal MTC. In the combination group, 8 MTC lesions showed extra-thyroidal extension (ETE), including 3 lesions that invaded the strap muscles, 2 lesions that invaded the internal jugular vein, and 1 lesion that invaded the recurrent laryngeal nerve. In the MTC group, 30 MTC lesions invaded the thyroid capsule, with 1 case being only microscopically invasive, 13 cases involving the strap muscles, and 25 cases involving other tissues (such as trachea, esophagus, internal jugular vein, recurrent laryngeal nerve). However, differences between two groups in multifocal and ETE were not significant (P > 0.05, **Table 2**).

**Table 2:** Pathology data of the MTC+PTC group and the MTC group.

	MTC+PTC group (n=25)	MTC group (n=134)	X² (t)	P
Maximum tumor diameter (mm)	20.00 ± 13.36	25.32 ± 14.94	1.408	0.162
Multiple lesions (n)			1.303	0.521
Yes	9 (36.0%)	44 (32.8%)		
No	9 (36.0%)	52 (38.8%)		
Thyroid membrane invasion (n)			0.683	0.409
Yes	8 (32.0)	30 (22.4%)		
No	17 (68.0)	104 (77.6%)		
Strap muscle invasion (n)			0.076	0.783
Yes	3 (12.0%)	13 (9.7%)		
No	22 (88.0%)	121 (90.3%)		
LN dissection (N)	41.17 ± 17.50	35.96 ± 20.77	-0.586	0.561
LN metastasis (N)	6.67 ± 6.69	11.69 ± 9.54	1.244	0.219
BCND (N)	7.05 ± 3.72	6.67 ± 5.33	-0.301	0.764
Metastasis of BCN (N)	1.39 ± 2.62	2.01 ± 2.92	0.844	0.401
ILND (N)	25.73 ± 14.71	30.62 ± 16.17	0.941	0.35
Metastasis of ILN (N)	2.00 ± 2.828	25.73 ± 14.37	1.601	0.114
pT (n)			8.022	0.431
T1	9 (50.0%)	34 (25.4%)		
T2	4 (16.0%)	25 (18.7%)		
T3	5 (20.0%)	25 (18.7%)		
T4	1 (4.0%)	16 (12.0%)		
Tx	4 (16.0%)	30 (22.4%)		
pN (n)			5.552	0.062
N0	6 (24.0%)	38 (28.4%)		
N1a	6 (24.0%)	11 (8.2%)		
N1b	6 (24.0%)	45 (33.6%)		
pM (n)			0.503	0.778
M0	22 (88.0%)	124 (92.5%)		
M1	1 (4.0%)	6 (4.5%)		
Mx	2 (8.0%)	2 (1.5%)		

MTC, Medullary thyroid carcinoma; PTC, Papillary thyroid carcinoma; LN, Lymph nodes; BCN, bilateral central lymph nodes; ILN, Ipsilateral lymph nodes.

There were no significant differences between the two groups in the number of LN resected, the number of LN metastasis, the maximum diameter of LN metastasis, and TNM staging (P > 0.05) (**Table 2**). Although the surgical approaches differed in proportion between the two groups, the difference was not significant (P > 0.05). The combination group primarily underwent TT+BCND+ILND, with other surgical approaches performed in only one case. Subgroup analysis revealed that no significant differences in the number of BCND or ILND and LN metastasis between the two groups.

### 3.3 Follow-up data comparison

In the combination group, 25 patients were followed up long-term, while 1 patient was lost to follow-up immediately post-surgery. In the MTC group, 8 patients were lost to follow-up, and the remaining patients were followed up. The mean follow-up period ranged from 3 to 80 months (34.3 ± 24.2 months) for the combination group, and from 2 to 87 months (29.4 ± 22.8 months) for the MTC group. Serum Ctn and CEA levels significantly decreased at 1 month post-operative in both groups compared to pre-operative levels, but the differences between the groups were not statistically significant (**Table 3**). At 1 and 6 month post-surgery, the thyroglobulin (Tg) level was higher in the combination group compared to the MTC group, but this difference did not reach significance. At the last follow-up, no significant differences in serum Ctn, CEA, and Tg levels were observed between the two groups.

**Table 3:** Follow-Up data of the MTC+PTC group and the MTC group.

	MTC+PTC group (n=25)	MTC group (n=134)	t	P
Pre-operative data (Median)				
Ctn (pg/mL)	80.53	272.8	0.346	0.73
CEA (ng/mL)	6.9	21.25	-0.079	0.937
Tg (ug/L)	12.6	12	0.728	0.47
Post-operative data (Median) (1 month)				
Ctn (pg/mL)	34.75	10.375	1.061	0.292
CEA (ng/mL)	4.555	6.34	-0.77	0.443
Tg (ug/L)	0.56	0.195	-0.603	0.551
Post-operative data (Median) (6 month)				
Ctn (pg/mL)	42.5	10.25	0.563	0.575
CEA (ng/mL)	1.795	2.12	-0.485	0.629
Tg (ug/L)	0.2	0.08	-0.761	0.455
Last follow-up data (Median)				
Ctn (pg/mL)	67.5	34.02	0.112	0.911
CEA (ng/mL)	3.83	2.64	-0.482	0.63
Tg (ug/L)	0.14	0.12	0.676	0.502

MTC, Medullary thyroid carcinoma; PTC, Papillary thyroid carcinoma; Ctn, Calcitonin; CEA, Carcinoembryonic antigen; Tg, Thyroglobulin.

### 3.4 Prognosis comparison

During the follow-up, 6 patients in the combination group were disease-free, 4 patients had biochemical abnormalities, 1 patient had cervical lymph node metastasis, and 1 patient had liver metastasis. In the MTC group, 65 patients were disease-free, 17 patients had biochemical abnormalities, and 11 patients had structural abnormalities. Among the 11 patients with structural abnormalities, 8 had cervical lymph node metastasis, 1 had lung metastasis, and 2 had metastases to the skull and ischium. All metastatic lesions were MTC metastases. The recurrence rate was similar between the two groups (P = 0.196).

### 3.5 Risk factors for biochemical/structural abnormalities

The univariate analysis revealed that the max tumor diameter (OR, 1.04; *P*=0.023), total tumor diameter (OR, 1.04; *P*=0.013), capsule invision (OR, 8.24; *P*=0.001), extracapsular invasion (OR, 24.7; *P*=0.003), recurrent nerve invasion and (OR, 14.48; *P*=0.014) were significantly associated with the risk of biochemical/structural abnormalities in MTC group (**Table 4**).

**Table 4:** Univariate logistic regression analysis for recurrence and metastasis in MTC and MTC+PTC group.

Variable	Recurrence in MTC group			Metastasis in MTC group			Recurrence in MTC+PTC group		
	OR/RR	95%CI	P	OR/RR	95%CI	P	OR/RR	95%CI	P
Gender (Female)	0.6	0.23~1.53	0.282	NA	NA	NA	0.43	0.03~4.99	0.486
Age	0.99	0.95~1.02	0.419	0.99	0.85~1.13	0.846	1.02	0.92~1.14	0.764
Tumor count	1.91	0.93~4.47	0.100	0.74	0.02~7.43	0.848	0.64	0.09~2.84	0.575
Max tumor diameter	1.04	1.01~1.08	0.023*	1.03	0.89~1.14	0.626	1	0.92~1.08	0.991
Total tumor diameter	1.04	1.01~1.07	0.013*	NA	NA	NA	0.98	0.88~1.07	0.669
Multifocal	1.68	0.63~4.55	0.299	NA	NA	NA	3	0.32~34.77	0.341
Capsule invision	8.24	2.58~32.3	0.001*	NA	NA	NA	5.25	0.53~72.52	0.172
Muscular involvement	NA	NA	NA	NA	NA	NA	5.33	0.38~144.17	0.232
Extracapsular invasion	24.7	4.44~464.99	0.003*	NA	NA	NA	NA	NA	NA
Recurrent nerve invasion	14.48	2.48~276.03	0.014*	NA	NA	NA	NA	NA	NA
Distant metastasis	2.27	0.21~50.23	0.513	NA	NA	NA	NA	NA	NA
Metastasis of BCN	0.97	0.82~1.15	0.742	1.26	0.68~2.14	0.342	NA	NA	NA
BCND	1	0.91~1.1	0.992	0.93	0.49~1.32	0.754	0.86	0.57~1.19	0.406
Metastasis of ILN	1.02	0.95~1.09	0.577	1.06	0.83~1.2	0.425	NA	NA	NA
ILND	1	0.98~1.03	0.923	1	0.86~1.09	0.955	NA	NA	NA
Max LN metastasis diameter	1.02	0.98~1.06	0.352	1.06	0.93~1.17	0.249	NA	NA	NA
Familial	0.94	0.21~3.86	0.929	NA	NA	NA	NA	NA	NA

MTC, Medullary thyroid carcinoma; PTC, Papillary thyroid carcinoma; BCN, bilateral central lymph nodes; BCND, bilateral central lymph node dissection; ILN, Ipsilateral lymph nodes; ILND, Ipsilateral lymph node dissection; OR, Odds ratio; RR, Risk ratio; 95% CI, 95% confidence interval.

*P<0.05.

Due to the missing of follow-up data, we only performed a multivariate analysis for recurrence in MTC group. In the multivariate analysis, only the max tumor diameter (OR, 1.04; *P*=0.042), and capsule invision (OR, 6.81; *P*=0.028) was independently associated with biochemical/structural abnormalities (**Table 5**).

**Table 5:** Multivariate logistic regression analysis for recurrence in MTC group.

Variable	OR	95%CI	P
Sex			
Male	Ref.		
Female	1.04	0.25-4.46	0.059
Age	0.95	0.89-1	0.96
Total tumor diameter	1.04	1-1.09	0.042*
Capsule invision	6.81	1.29-43.05	0.028*
Extracapsular invasion	7.89	0.77-191.15	0.112

RR, Risk ratio; 95% CI, 95% confidence interval.

*P<0.05.

## 4 Discussion

Currently, the etiology of patients with concurrent MTC and PTC is not well clarified. There are five main hypotheses regarding the co-occurrence of MTC and PTC (18). The first is the hostage theory (19, 20), which primarily explains mixed MTC-PTC cases. Microscopic analysis has revealed that MTC can entrap follicular cells, leading to hyperplastic follicular foci. Genetic mutations during proliferation may result in neoplastic transformation. The second is the local effect hypothesis (21, 22), which asserts that factors such as radiation can induce tumors of both cellular origins simultaneously. The third is the stem cell hypothesis (23), based on the idea that both follicular epithelial and parafollicular cells share common proto-oncogenes. The fourth is the different differentiation theory (12), which posits that both types of cells activate shared oncogenic pathways. The fifth is the collision theory (24), which proposes that the simultaneous occurrence of two tumors is merely coincidental, given the higher incidence of PTC among thyroid cancers.

A search of the literature yielded only four studies analyzing this phenomenon, with three supporting the collision hypothesis (11, 16, 25) and one supporting the stem cell hypothesis (26). Kim et al. (16) reported that the combined group was older than the MTC-only group (53.5 years vs. 44.5 years, P=0.009), but this finding was not corroborated by other literatures or by our study. Machens et al. (25) noted that the maximum diameter of MTC in the combined group was smaller (11 mm vs. 20 mm, P=0.008), suggesting that more meticulous pathological examination is required to detect PTC lesions. However, neither Kim et al. nor our study found a significant difference in MTC maximum diameter between the groups. Machens et al. also observed that the incidence of MTC combined with PTC was lower in Germany compared to Italy and Korea (3.6% vs. 13.8% vs. 18.9%, respectively), which may be due to higher iodine levels in the latter two regions. In areas with higher iodine levels such as Chengdu (27), the proportion of PTC in the thyroid cancer pathology is relatively higher. In our study, the incidence of MTC combined with PTC was 15.7%. In addition, Shifrin et al. (26) suggested that MTC patients with RET-V804M mutations are more likely to have concomitant PTC. However, Machens et al. (25) reported that RET-V804M mutation-positive MTC patients did not detect PTC. Although our study did not include genetic testing, current research does not establish a definitive correlation between RET gene mutations and co-existence of MTC and PTC. Future multi-center studies with systematic genetic testing are warranted to further elucidate the potential role of RET mutations in the co-occurrence of MTC and PTC. Given the conflicting reports regarding RET mutations and their association with PTC co-occurrence, the lack of genetic data in our study limits our ability to assess whether genetic background influences the clinicopathological features or outcomes observed in our cohort. However, our findings on prognosis and tumor behavior remain clinically relevant, as they reflect real-world scenarios where genetic testing is not always routinely performed. Future multi-center studies integrating comprehensive molecular profiling with clinical data are needed to determine whether specific genetic alterations modify the biological behavior of concurrent MTC and PTC. Biscolla et al. (11) classified the prognosis of MTC patients and indicated that PTC does not affect the prognosis of MTC, a conclusion that is consistent with our findings.

In this study, all patients had independent cancer foci, with 76% (19/25) having the two cancer foci located in different lobes, thus not supporting the hostage theory. There were no significant differences in demographic characteristics, pathological data, follow-up data, or prognosis between the two groups. This suggests that the presence of PTC does not significantly alter the invasiveness or prognosis compared to isolated MTC. However, it is important to note that more extensive surgical interventions (e.g., total thyroidectomy with central lymph node dissection) may be required in cases of concurrent MTC and PTC, potentially increasing the risk of postoperative complications such as hypocalcemia and recurrent laryngeal nerve injury. Previous studies have highlighted that demolitive thyroid surgery carries higher rates of transient or permanent hypocalcemia, particularly in patients without adequate calcium and vitamin D supplementation (28). Additionally, completion thyroidectomy after initial hemithyroidectomy may pose additional risks, including vocal cord dysfunction (29). Preoperative assessment of vocal cord motility using transcutaneous laryngeal ultrasonography could help mitigate this risk (30). The lack of consistent differences in clinical data between the combined group and the MTC group indicates that observed differences might be due to random factors. Considering all available evidence, while the local effect hypothesis, stem cell hypothesis, and different differentiation hypothesis cannot be entirely excluded, the collision hypothesis seems to be more plausible.

Of course, this study had several limitations. First, our study was retrospective trials, which inevitably add a degree of selection bias to the results. Second, despite having 25 patients in the combined group, which is larger compared to previous case reports, relatively small number of patients with short-term follow-up data were included in our study. Third, due to financial constraints, patients were reluctant to detect the RET proto-oncogene mutation. Accordingly, well-designed trials with larger volume and longer follow-up are needed to confirm the mechanisms and relationships of MTC combined with PTC.

In conclusion, this study reveals no significant difference in the invasiveness and prognosis between the MTC-PTC co-existence and only MTC groups.

## Data Availability

The original contributions presented in the study are included in the article/Supplementary Material. Further inquiries can be directed to the corresponding authors.

## References

[1] Jung KW Won YJ Kong HJ Oh CM Seo HG Lee JS. Cancer statistics in Korea: incidence, mortality, survival and prevalence in 2010. Cancer Res Treat. (2013) 45:1–14. doi: 10.4143/crt.2013.45.1.1, PMID: 23613665 PMC3629358

[2] Lim H Devesa SS Sosa JA Check D Kitahara CM. Trends in thyroid cancer incidence and mortality in the United States, 1974-2013. Jama. (2017) 317:1338–48. doi: 10.1001/jama.2017.2719, PMID: 28362912 PMC8216772

[3] Pizzato M Li M Vignat J Laversanne M Singh D La Vecchia C. The epidemiological landscape of thyroid cancer worldwide: GLOBOCAN estimates for incidence and mortality rates in 2020. Lancet Diabetes Endocrinol. (2022) 10:264–72. doi: 10.1016/S2213-8587(22)00035-3, PMID: 35271818

[4] Boucai L Zafereo M Cabanillas ME. Thyroid cancer: A review. Jama. (2024) 331:425–35. doi: 10.1001/jama.2023.26348, PMID: 38319329

[5] Chen DW Lang BHH McLeod DSA Newbold K Haymart MR. Thyroid cancer. Lancet. (2023) 401:1531–44. doi: 10.1016/S0140-6736(23)00020-X, PMID: 37023783

[6] Cabanillas ME McFadden DG Durante C. Thyroid cancer. Lancet. (2016) 388:2783–95. doi: 10.1016/S0140-6736(16)30172-6, PMID: 27240885

[7] Nabhan F Dedhia PH Ringel MD. Thyroid cancer, recent advances in diagnosis and therapy. Int J Cancer. (2021) 149:984–92. doi: 10.1002/ijc.33690, PMID: 34013533

[8] Seib CD Sosa JA. Evolving understanding of the epidemiology of thyroid cancer. Endocrinol Metab Clin North Am. (2019) 48:23–35. doi: 10.1016/j.ecl.2018.10.002, PMID: 30717905

[9] Pelizzo MR Mazza EI Mian C Merante Boschin I. Medullary thyroid carcinoma. Expert Rev Anticancer Ther. (2023) 23:943–57. doi: 10.1080/14737140.2023.2247566, PMID: 37646181

[10] Papotti M Negro F Carney JA Bussolati G Lloyd RV. Mixed medullary-follicular carcinoma of the thyroid. A morphological, immunohistochemical and in *situ* hybridization analysis of 11 cases. Virchows Arch. (1997) 430:397–405. doi: 10.1007/s004280050049, PMID: 9174630

[11] Biscolla RP Ugolini C Sculli M Bottici V Castagna MG Romei C. Medullary and papillary tumors are frequently associated in the same thyroid gland without evidence of reciprocal influence in their biologic behavior. Thyroid. (2004) 14:946–52. doi: 10.1089/thy.2004.14.946, PMID: 15671773

[12] Younes N Shomaf M Al Hassan L. Simultaneous medullary and papillary thyroid carcinoma with lymph node metastasis in the same patient: case report and review of the literature. Asian J Surg. (2005) 28:223–6. doi: 10.1016/S1015-9584(09)60348-1, PMID: 16024321

[13] Fibbi B Pinzani P Salvianti F Rossi M Petrone L De Feo ML. Synchronous occurrence of medullary and papillary carcinoma of the thyroid in a patient with cutaneous melanoma: determination of BRAFV600E in peripheral blood and tissues. Rep Case Rev Literature Endocr Pathol. (2014) 25:324–31. doi: 10.3390/cancers14102472, PMID: 24858900

[14] Fallahi P Patrizio A Stoppini G Elia G Ragusa F Paparo SR. Simultaneous occurrence of medullary thyroid carcinoma and papillary thyroid carcinoma: A case series with literature review. Curr Oncol. (2023) 30:10237–48. doi: 10.3390/curroncol30120745, PMID: 38132379 PMC10742226

[15] Appetecchia M Lauretta R Barnabei A Pieruzzi L Terrenato I Cavedon E. Epidemiology of simultaneous medullary and papillary thyroid carcinomas (MTC/PTC): an Italian multicenter study. Cancers (Basel). (2019) 11(10). doi: 10.3390/cancers11101516, PMID: 31600997 PMC6826384

[16] Kim WG Gong G Kim EY Kim TY Hong SJ Kim WB. Concurrent occurrence of medullary thyroid carcinoma and papillary thyroid carcinoma in the same thyroid should be considered as coincidental. Clin Endocrinol (Oxf). (2010) 72:256–63. doi: 10.1111/j.1365-2265.2009.03622.x, PMID: 20447064

[17] Wells SA Jr. Asa SL Dralle H Elisei R Evans DB Gagel RF. Revised American Thyroid Association guidelines for the management of medullary thyroid carcinoma. Thyroid. (2015) 25:567–610. doi: 10.1089/thy.2014.0335, PMID: 25810047 PMC4490627

[18] Bojoga A Stănescu L Badiu C. Collision tumors of the thyroid. A special clinical and pathological entity. Arch Clin cases. (2021) 8:84–90. doi: 10.22551/2021.33.0804.10191, PMID: 34984231 PMC8717004

[19] Volante M Papotti M Roth J Saremaslani P Speel EJ Lloyd RV. Mixed medullary-follicular thyroid carcinoma. Molecular evidence for a dual origin of tumor components. Am J Pathol. (1999) 155:1499–509. doi: 10.1016/S0002-9440(10)65465-X, PMID: 10550306 PMC1866972

[20] Gurkan E Gurbuz Y Tarkun I Canturk Z Cetinarslan B. Mixed medullary-papillary carcinoma of the thyroid: report of two cases and review of the literature. Indian J Pathol Microbiol. (2014) 57:598–602. doi: 10.4103/0377-4929.142684, PMID: 25308015

[21] Triggs SM Williams ED. Experimental carcinogenesis in the thyroid follicular and C cells. A comparison of the effect of variation in dietary calcium and of radiation. Acta Endocrinol (Copenh). (1977) 85:84–92., PMID: 577082

[22] Ciampi R Romei C Pieruzzi L Tacito A Molinaro E Agate L. Classical point mutations of RET, BRAF and RAS oncogenes are not shared in papillary and medullary thyroid cancer occurring simultaneously in the same gland. J Endocrinol Invest. (2017) 40:55–62. doi: 10.1007/s40618-016-0526-5, PMID: 27535135

[23] Ljungberg O Ericsson UB Bondeson L Thorell J. A compound follicular-parafollicular cell carcinoma of the thyroid: a new tumor entity? Cancer. (1983) 52:1053–61. doi: 10.1002/1097-0142(19830915)52:6<1053::AID-CNCR2820520621>3.0.CO;2-Q, PMID: 6136320

[24] Pastolero GC Coire CI Asa SL. Concurrent medullary and papillary carcinomas of thyroid with lymph node metastases. A collision phenomenon. Am J Surg Pathol. (1996) 20:245–50. doi: 10.1097/00000478-199602000-00014, PMID: 8554115

[25] Machens A Dralle H. Simultaneous medullary and papillary thyroid cancer: a novel entity? Ann Surg Oncol. (2012) 19:37–44. doi: 10.1245/s10434-011-1795-z, PMID: 21626080

[26] Shifrin AL Xenachis C Fay A Matulewicz TJ Kuo YH Vernick JJ. One hundred and seven family members with the rearranged during transfection V804M proto-oncogene mutation presenting with simultaneous medullary and papillary thyroid carcinomas, rare primary hyperparathyroidism, and no pheochromocytomas: is this a new syndrome–MEN 2C? Surgery. (2009) 146:998–1005. doi: 10.1016/j.surg.2009.09.021, PMID: 19958926

[27] Shan Z Chen L Lian X Liu C Shi B Shi L. Iodine status and prevalence of thyroid disorders after introduction of mandatory universal salt iodization for 16 years in China: A cross-sectional study in 10 cities. Thyroid. (2016) 26:1125–30. doi: 10.1089/thy.2015.0613, PMID: 27370068

[28] Tolone S Roberto R del Genio G Brusciano L Parmeggiani D Amoroso V. The impact of age and oral calcium and vitamin D supplements on postoperative hypocalcemia after total thyroidectomy. A prospective study. BMC Surg. (2013) 13 Suppl 2:S11. doi: 10.1186/1471-2482-13-S2-S11, PMID: 24267491 PMC3851172

[29] Canu GL Medas F Cappellacci F Giordano ABF Gurrado A Gambardella C. Risk of complications in patients undergoing completion thyroidectomy after hemithyroidectomy for thyroid nodule with indeterminate cytology: an Italian multicenter retrospective study. Cancers (Basel). (2022) 14(10). doi: 10.3390/cancers14102472, PMID: 35626075 PMC9139447

[30] Gambardella C Offi C Romano RM De Palma M Ruggiero R Candela G. Transcutaneous laryngeal ultrasonography: a reliable, non-invasive and inexpensive preoperative method in the evaluation of vocal cords motility-a prospective multicentric analysis on a large series and a literature review. Updates Surg. (2020) 72:885–92. doi: 10.1007/s13304-020-00728-3, PMID: 32124271

